# Assessment of renal function and electrolytes in patients with thyroid dysfunction in Addis Ababa, Ethiopia: a cross sectional study

**DOI:** 10.11604/pamj.2016.24.338.8455

**Published:** 2016-08-31

**Authors:** Nardos Abebe, Tedla Kebede, Mistire Wolde

**Affiliations:** 1 Bethzatha Advanced Medical Laboratory and Registered Assessor of ISO 15189, Addis Abeba, Ethiopia; 2 Department of Internal medicine, College of Health Science, Addis Abeba University, Ethiopia; 3 Department of Medical Laboratory Sciences, College of Health Science, Addis Abeba University, Ethiopia

**Keywords:** Thyroid dysfunction, renal function tests, blood electrolyte

## Abstract

**Introduction:**

Studies demonstrated that abnormal thyroid functions may result in decreased or increased kidney size, kidney weight, and affect renal functions. In this regard, studies on the association of abnormal thyroid functions and renal function tests are scarcely found in Ethiopia.

**Objective:**

To assess renal function and electrolytes in patients with thyroid dysfunction, in Addis Ababa, Ethiopia.

**Methods:**

Cross sectional study was conducted from March 21/2015-May 27/2015 at Arsho Advanced Medical Laboratory. During the study period, 71 patients with thyroid dysfunction were eligible, and socio demographic data collected by structured questionnaire. Then blood sample was collected for thyroid function tests, renal function and blood electrolyte analysis. The collected data was analyzed by SPSS version 20. ANOVA and binary logistic regression were employed to evaluate the mean deference and associations of thyroid hormone with renal function and electrolyte balances.

**Results:**

Among the renal function tests, serum uric acid, and creatinine mean values were significantly decreased in hyperthyroid patients; whereas, eGFR mean value was significantly increased in hyperthyroid study patients (P<0.05). Meanwhile, from the electrolyte measurements made, only the mean serum sodium value was significantly increased in hyperthyroid study participants. Binary logistic regression analysis on the association of thyroid dysfunction with electrolyte balance and renal function tests indicated that serum sodium, creatinine, eGFR values and hyperthyroidism have a statistical significant association at AOR 95% CI of 0.141(0.033-0.593, P=0.008); 16.236(3.481-75.739, P=0.001), and 13.797(3.261-58.67, P=0.001) respectively.

**Conclusion:**

The current study reveals, thyroid abnormalities may lead to renal function alterations and also may disturb electrolyte balance. Knowledge of this significant association has worthwhile value for clinicians, to manage their patients' optimally.

## Introduction

The interplay between thyroid and kidney in each other's function are known for many years [1]. Thyroid dysfunction affects renal physiology and development, whereas kidney failure could result in thyroid dysfunction [1]. Abnormalities in thyroid hormones synthesis, secretion, or actions usually categorized as hypothyroidism, and hyperthyroidism, depending on the concentration of T3 and T4 in the circulation. This thyroid function abnormalities may be associated with different complication, including disturbances in the renal function and electrolyte balance [2–5]. Hypothyroidism is a biochemical and/or clinical disorder characterized by deficiency of thyroid hormones which, in turn, results in a generalized slowing down of metabolic processes [6]. It is associated with many biochemical abnormalities including increased serum creatinine, and uric acid [7]. Moreover, functional changes including reduced renal blood flow, glomerular filtration rate and absorption of sodium, chloride and water are seen [5, 8]. On the contrary, hyperthyroidism is a biochemical and clinical entity resulting from the hyper secretion of thyroid hormones which, in turn, results in a generalized hyper activation of metabolic processes [2]. Moreover, Hyperthyroidism can result in or accelerate chronic kidney disease (CKD) by several mechanisms. Firstly, hyperthyroidism results in intra- glomerular hypotension (increased filtration pressure) and consequent hyper-filtration. Secondly, hyperthyroidism predisposes to proteinuria, which is known to cause direct renal injury. Thirdly, hyperthyroidism-induced mitochondrial energy metabolism along with down- regulation of superoxide dismutase contributes to the increased free radical generation and consequent renal injury [1, 9]. Conversely, hypothyroidism doesn't contribute to progression of CKD except by mild to moderate reduction in GFR [1]. While the effects of thyroid hormones on renal functions are well known, the effects on electrolytes have not been well studied. Moreover, the effects of thyroid hormones on renal function and electrolyte balances have not been well established for Ethiopian population. With this background, the present study was undertaken to assess the renal function and alterations in the level of serum electrolytes in patients with thyroid dysfunction.

## Methods

Cross sectional study was conducted on 71 thyroid dysfunction patient's attending at Arsho Advanced Medical Laboratory from March 21/2015 to May 26/2015. Clinical data was obtained from the participants' history and recorded on a questionnaire sheet. Clinical assessment of the study group was done by clinicians and they were not suffering from other disorder like renal failure, cardiac problem and hypertension. After 12 hours overnight fasting, 4ml of blood was drawn from all the study participants by venipuncture. Samples were centrifuged at 3000RPM for 10 min. T3, T4 and TSH were measured by chemiluminescence immunoassay method on Beckman Coulter Access 2 auto analyzer. Renal function tests (RFT) including serum creatinine (Cr), blood urea nitrogen (BUN), uric acid (UA), sodium (Na), Potassium (K), and estimated Glomerular Filtration Rate (eGFR) values, and as well electrolyte tests including K, Na, Calcium (Ca), and Chloride (Cl) values were analyzed on Beckman Coulter AU-680 auto analyzer. Statistical Analysis All the data obtained were entered in to SPSS version 20 software. ANOVA was used to assess the mean differences of renal function tests and electrolyte balances. Binary logistic regression was used to assess the association of thyroid hormones with renal function and electrolyte balance test. Also TSH mean values were calculated. Finally a P value of < 0.05 was considered as significant.

## Results

Among the total 71 study participants the proportion of Euthyroid patients was relatively high. Moreover of the study participants were females, and mean age of the study participants were 41.9±9.7years of age, as shown in Table 1. As well, mean concentrations of TSH values were shown in Table 2. Of the total RFT test parameters, the mean concentration of BUN, and eGFR were higher in hyperthyroid patients. However, the mean concentrations of all Cr, UA, except, eGFR, in hyperthyroid participant, were within the physiological reference range, all the mean concentrations statistically differed significantly (P< 0.05) when compared to euthyroid participants as shown in Table 3. Likewise, from the measured serum electrolytes, mean concentrations of Na, Cl and Ca were higher in hyperthyroid participants which were shown in Table 4. Binary logistic regression analysis demonstrated that the mean concentrations of Na, Cr and eGFR were significantly associated with hyperthyroid cases. These associations of hyperthyroidism and the selected abnormal renal function and electrolyte balance were not significantly changed after adjusting the sex and age. As shown in Table 5.

**Table 1:** Sex and age category of study participants with thyroid dysfunction attending at Arsho Advanced Medical Laboratory, Addis Ababa, Ethiopia

Variable		Thyroid status		
		Hypothyroid(n=25)35%	Euthyroid(n=29) 41%	Hyperthyroid(n=17)24%
Age	18-27	1(1.4%)	3(4.2%)	1(1.4%)
	28-37	6(8.5%)	5(7.0%)	6(8.5%)
	38-47	10(13.8%)	8(11.3%)	7(9.9%)
	48-60	8(11.3%)	13(18.5%)	3(4.2%)
Sex	Male	5(7.0%)	5(7.0%)	7(9.9%)
	Female	24(33.8%)	12(16.9%)	18(25.4%)

**Table 2:** Mean concentration values of serum TSH among study participants attending at Arsho Advanced Medical Laboratory, Addis Ababa, Ethiopia

Thyroid dysfunction	TSH mean value	TSH Reference Range
Euthyroid	1.44	0.34-5.6
Hyperthyroid	0.046	
Hypothyroid	28.4	

**Table 3:** Mean concentration of renal function test parameters among thyroid dysfunction patients attending at Arsho Advanced Medical Laboratory

Type of thyroid abnormalities				
Renal functon	Hypothyroid (Mean±SD)	Euthyroid (Mean±SD)	Hyperthyroid (Mean±SD)	P value
Urea	22.9±9.3mg/dl	21.9±10.6 mg/dl	24.2±14.7 mg/dl	0.911
Creatinine	0.7±0.07mg/dl	0.8±0.2 mg/dl	0.6±0.3 mg/dl	0.001
Uric Acid	4.4±1.1mg/dl	5.5±1.9 mg/dl	4.9±1.4 mg/dl	0.042
eGFR	136.5±28.3ml/min	117.8±41.6ml/min	202.2±103.9ml/min	0.001

**Table 4:** Mean concentration of serum electrolyte with respect to thyroid dysfunction, among study participants attending at Arsho Advanced Medical Laboratory, Addis Ababa, Ethiopia

Type of thyroid abnormalities				
Electrolytebalance(Renal function)	Hypothyroid(Mean±SD)	Euthyroid(Mean±SD)	Hyperthyroid(Mean±SD)	P-value
Sodium	135.7±3.6mmol/L	136.2±3.2 mmol/L	138.5±2.3 mmol/L	0.016
Chloride	103.8±3.5mmol/L	104.2±2.7 mmol/L	106.4±4.0 mmol/L	0.377
Calcium	9.3±0.5mmol/L	9.2±0.5 mmol/L	9.5±0.5 mmol/L	0.474
Potassium	4.2±0.4mmol/L	4.4±0.8 mmol/L	4.3±0.6 mmol/L	0.391

**Table 5:** Unadjusted and adjusted effect of thyroid dysfunction on the electrolyte balance and renal function tests of the study participants with thyroid dysfunction attending at Arsho Advanced Medical Laboratory, Addis Ababa, Ethiopia.

	Hyperthyroid		Hypothyroid	
Variables	UAOR(95% CI)	AOR(95% CI)	UAOR(95% CI)	AOR(95% CI)
Sodium	0.146(0.036-0.598)	0.141(0.033-0.593^¥^	0.584(0.170-2.085)	0.532(.144-1.967)
Uric acid	1.182(0.216-6.457)	1.008(0.162-6.259)	3.611(0.739-17.644)	3.466(0.596-20.172)
Creatinine	13.282(3.446-51.187)	16.236(3.481-75.739)^€^	1.923(0.413-8.965)	1.189(0.215-6.569)

¥ P< 0.05

€ P< 0.001

## Discussion

The association of thyroid dysfunction and abnormal renal function tests has been demonstrated in different studies. On the current study hyperthyroid patient's mean values of creatinine, and uric acid were significantly decreased; while eGFR value was significantly increased. Moreover, Serum creatinine, and eGFR mean concentration values have a significant association with hyperthyroidism. On the other hand, from the assessed serum electrolyte, sodium value showed a significant increment in hyperthyroid subjects. Hyperthyroidism is associated with decreased mean values of serum creatinine, and uric acid. Whereas eGFR, serum sodium and urea mean values indicate an increment. Similar changes in eGFR, Urea and Creatinine have been reported in few studies involving different number of hyperthyroid subjects [10, 11]. When there is high concentration of thyroid hormones, it will increase the cardiac output by positive chronotropic and introtropic effects. As a result of this, GFR increases, [12, 13] and consequently, serum creatinine value decreased [1]. Moreover, for the increment of eGFR in hyperthyroid cases, Insulin like growth factor type-I [14, 15] and activation of rennin- angotensin- aldosterone system (RAAS) [1] also have contributions. In the present study serum uric acid was significantly decreased in the study participants with hyperthyroidism. These study findings was in contrasts with research conducted by Gulabkanwar et al 2014, where hyper secretion of uric acid attributed to increased level of thyroid hormones (T3 and T4), which cause increase rate of the metabolites such as purine, which in turn increased production of uric acid in the blood and exceed the renal capacity to excrete uric acid [16]. On contrary to the finding of Gulabkanwar et al, hyper secretion of T3 and T4 results in increased GFR and renal plasma flow. Consequently, serum uric acid value decreased [1]. Hypothyroidism, another main type of thyroid dysfunction assessed on the current study, has been associated with decrease mean values of creatinine and uric acid, and increase mean value of eGFR. The decrement of serum creatinine in the present study as well as other studies [8, 17] argues against the previously held notion of increased serum creatinine value due to a decrease in eGFR or alteration in RAAS. But in the current study, increased eGFR and decreased serum creatinine values might be due to, abnormality in creatin kinase level muscular weakness, dystrophy, and poor physiological activity, or in agreement with Kreisman SH, et al, it might be due to the net unchanged creatinine value due to a balance between the decrease in renal clearance and a decrease in creatinine generation [18]. In the current study serum uric acid is significantly decreased in study participant with hypothyroidism, which contrasts with that obtained by Nagarajappa K.J, et al 2012 [8]. Hypo secretion characterized by decreased thyroid hormones level (T3 and T4) resulting in Deficiencies of these hormones cause significant changes in metabolism such as decrease in purine metabolism. The cause of decreased uric acid is believed to be mainly due to decreased metabolic activity in hypothyroidism. The current study also demonstrated that hyperthyroidism was associated with increase Serum Sodium values, whereas calcium, Chloride, and potassium mean values were not changed. As far as the investigators' knowledge, scarce research was conducted before on electrolyte balance and hyperthyroidism. This significant increment of sodium ions in the case of hyperthyroidism could possibly be due to availability of increased thyroid hormones, the Na-H exchanger and Na-Pico-transporter activity will also increase first in proximal tubules then almost all segments of nephron [19–21]. Another possibility for the increment of serum sodium value might be of the direct relation of hyperthyroidism with plasma rennin activity, and plasma level of angiotensinogen, angitension II and aldosterone [1]. In the case of hypothyroidism, almost all the assessed electrolyte balance indicates a decrement; however the decrement is not statistically significant. This study finding was different from similar studies conducted by Chaudhury HS et al [22]. Decreased thyroid hormones, decreases the plasma rennin, angiotensin II, and serum angiotensin converting enzyme levels. In addition, there is a net decrease in the RAAS activity. This results in afferent arteriolar vasoconstriction and efferent arteriolar vasodilatation. This could result in hypo perfusion of proximal convoluted tubule (PCT) and consequent lukewarm Na and Cl reabsorption in PCT. In addition, there is a decreased activity of basolateral NA/K ATPase, apical Na-H exchanger (NHE), and the Na-Pi co-transporter. Deactivation of these transporters decreases the proximal reabsorption. [1, 5, 19–21].

## Conclusion

The present study demonstrated that hypothyroidism and hyperthyroidism are associated with significant changes in renal function and electrolyte balance, despite being within the biological reference range. Although the number of study participants, and test parameters are limited, the current study findings give a clue that thyroid abnormalities may lead to not only renal function changes but also change in the serum electrolyte values for these Ethiopian study population. Owing to the small sample size of our study, it may not be appropriate to conclude that these statistically significant changes in the renal function and serum electrolyte which are within the physiological reference range are clinically significant to impact patient outcomes. Therefore, further studies with larger sample sizes are needed to see if routine follow-up of the renal function tests and serum electrolyte will improve the clinical outcomes of the patients with thyroid dysfunction.

### What is known about this topic

- Association between hypothyroidism and renal function;
- Association between hyperthyroidism and renal function.

### What this study adds

- Association between electrolyte and thyroid function;
- Effect of thyroid abnormality on renal function and electrolyte balance on Ethiopian population.
